# Osteoporosis in nontuberculous mycobacterial pulmonary disease: a cross-sectional study

**DOI:** 10.1186/s12890-022-01991-3

**Published:** 2022-05-21

**Authors:** Hiromu Tanaka, Takanori Asakura, Shoji Suzuki, Satoshi Okamori, Tatsuya Kusumoto, Takunori Ogawa, Shunsuke Uno, Atsuho Morita, Ho Lee, Ho Namkoong, Hirofumi Kamata, Yasunori Sato, Yoshifumi Uwamino, Tomoyasu Nishimura, Makoto Ishii, Koichi Fukunaga, Naoki Hasegawa

**Affiliations:** 1 Division of Pulmonary Medicine, Department of Medicine, Keio University School of Medicine, 35 Shinanomachi, Shinjuku, Tokyo, 160-8582 Japan; 2 Department of Infectious Diseases, Keio University School of Medicine, 35 Shinanomachi, Shinjuku, Tokyo, 160-8582, Japan; 3 Department of Preventive Medicine and Public Health, Keio University School of Medicine, 35 Shinanomachi, Shinjuku, Tokyo, 160-8582 Japan; 4 Department of Laboratory Medicine, Keio University School of Medicine, 35 Shinanomachi, Shinjuku, Tokyo, 160-8582 Japan; 5 Keio University Health Center, Tokyo, Japan

**Keywords:** Nontuberculous mycobacteria (NTM), *Mycobacterium avium* complex (MAC), Sex hormone, Postmenopausal women, The Lady Windermere's syndrome

## Abstract

**Background:**

Since nontuberculous mycobacterial pulmonary disease (NTM-PD) is common in middle-aged/elderly slender women at risk of osteoporosis, we hypothesized that NTM-PD could be associated with osteoporosis. The study aimed to evaluate the prevalence of osteoporosis in patients with NTM-PD compared with that in the general population and determine the factors associated with osteoporosis in the subjects, including the serum estradiol (E_2_) and 25-hydroxyvitamin D (25OHD) levels.

**Methods:**

We have recruited 228 consecutive adult patients with NTM-PD from a prospective cohort study at the Keio University Hospital, who had no history of osteoporosis or osteoporosis-associated bone fracture but underwent dual-energy X-ray absorptiometry-based bone mineral density (BMD) evaluation from August 2017–September 2019. The E_2_ and 25OHD levels were measured in 165 patients with available stored serum samples. We performed multivariable logistic regression analyses for osteopenia and osteoporosis.

**Results:**

Osteoporosis (T-score ≤  − 2.5) and osteopenia (T-score − 1 to − 2.5) were diagnosed in 35.1% and 36.8% of patients with NTM-PD, respectively. Compared with the general population, the proportion of osteoporosis was significantly higher in 50–59-, 60–69-, and 70–79-year-old women with NTM-PD. Multivariable analysis revealed that older age (adjusted odds ratio [aOR] for 1-year increase = 1.12; 95% confidence interval [CI] = 1.07–1.18), female sex (aOR = 36.3; 95% CI = 7.57–174), lower BMI (aOR for 1 kg/m^2^ decrease = 1.37; 95% CI = 1.14–1.65), and chronic *Pseudomonas aeruginosa* (PA) infection (aOR = 6.70; 95% CI = 1.07–41.8) were independently associated with osteoporosis. Additionally, multivariable analysis in 165 patients whose serum E_2_ and 25OHD levels were measured showed that both low E_2_ levels (< 10 pg/mL) and lower 25OHD levels were independently associated with osteoporosis.

**Conclusions:**

Middle-aged/elderly women with NTM-PD have a higher prevalence of osteoporosis than the general population. BMD screening should be considered in NTM-PD, especially in older females with severe diseases such as chronic PA infection and lower BMI, and low serum E_2_ and 25OHD levels.

**Supplementary Information:**

The online version contains supplementary material available at 10.1186/s12890-022-01991-3.

## Background

Nontuberculous mycobacterial (NTM) pulmonary disease (NTM-PD) is the most common NTM infection, and its incidence and prevalence have increased worldwide. A Japanese epidemiological study reported that there were 14.7 estimated cases of NTM-PD per 100,000 person-years in 2014 [1]. The characteristics of patients with NTM-PD differ between Europe and Japan; there are more males and a higher frequency of chronic obstructive pulmonary disease complications in Europe compared with that in Japan [2–4]. The progression of NTM-PD leads to decreased lung function, decreased health-related quality of life, and a poorer prognosis [5–7]. Progressive NTM-PD requires prolonged multidrug antimicrobial therapy; however, macrolide resistance and high recurrence rate may make the management difficult [8]. Thus, as NTM-PD often presents as a chronic disease, physicians should pay attention to comorbidities while managing it.

NTM-PD commonly occurs in middle-aged/elderly, slender, and postmenopausal women [9], and this population also presents with risk factors for osteoporosis, which is a systemic skeletal disease leading to increased bone fragility and fracture risk [10]. Our prior study showed that low serum estradiol (E_2_) levels were strongly associated with *Mycobacterium avium* complex (MAC) pulmonary disease (MAC-PD) [11]. Another study showed that NTM-PD is associated with severe vitamin D deficiency [12], which can lead to osteoporosis. Moreover, previous studies on chronic respiratory diseases, such as chronic obstructive pulmonary disease (COPD), idiopathic pulmonary fibrosis, and bronchiectasis without NTM infection, have shown to be associated with osteoporosis [13–15]. Some chronic lung diseases, especially those that cause structural destruction of the lungs, such as idiopathic pulmonary fibrosis [14] and bronchiectasis [15], have been reported to be associated with osteoporosis [16]. Patients with COPD are also reported to be at a higher risk of osteoporosis and fractures compared with age- and sex-matched patients without COPD [17]. Therefore, we hypothesized that NTM-PD could be associated with osteoporosis, especially when associated with low serum E_2_ and 25-hydroxyvitamin D (25OHD) levels.

Two claims-data-based analyses showed that osteoporosis in NTM-PD was one of the most common comorbidities [18] and had a higher prevalence than that in sex-and age-matched controls [3]. Another study with MAC-PD reported lower bone mineral density (BMD) of the thoracic and lumbar vertebrae [19]. However, there have been no reports on the prevalence and factors associated with osteoporosis in patients with NTM-PD in clinical settings. We aimed to evaluate the prevalence of osteoporosis in patients with NTM-PD compared with that in the general population and determine the factors associated with osteoporosis in the subjects, including the serum E_2_ and 25OHD levels.

## Methods

### Study design and patients

**Fig. 1:**
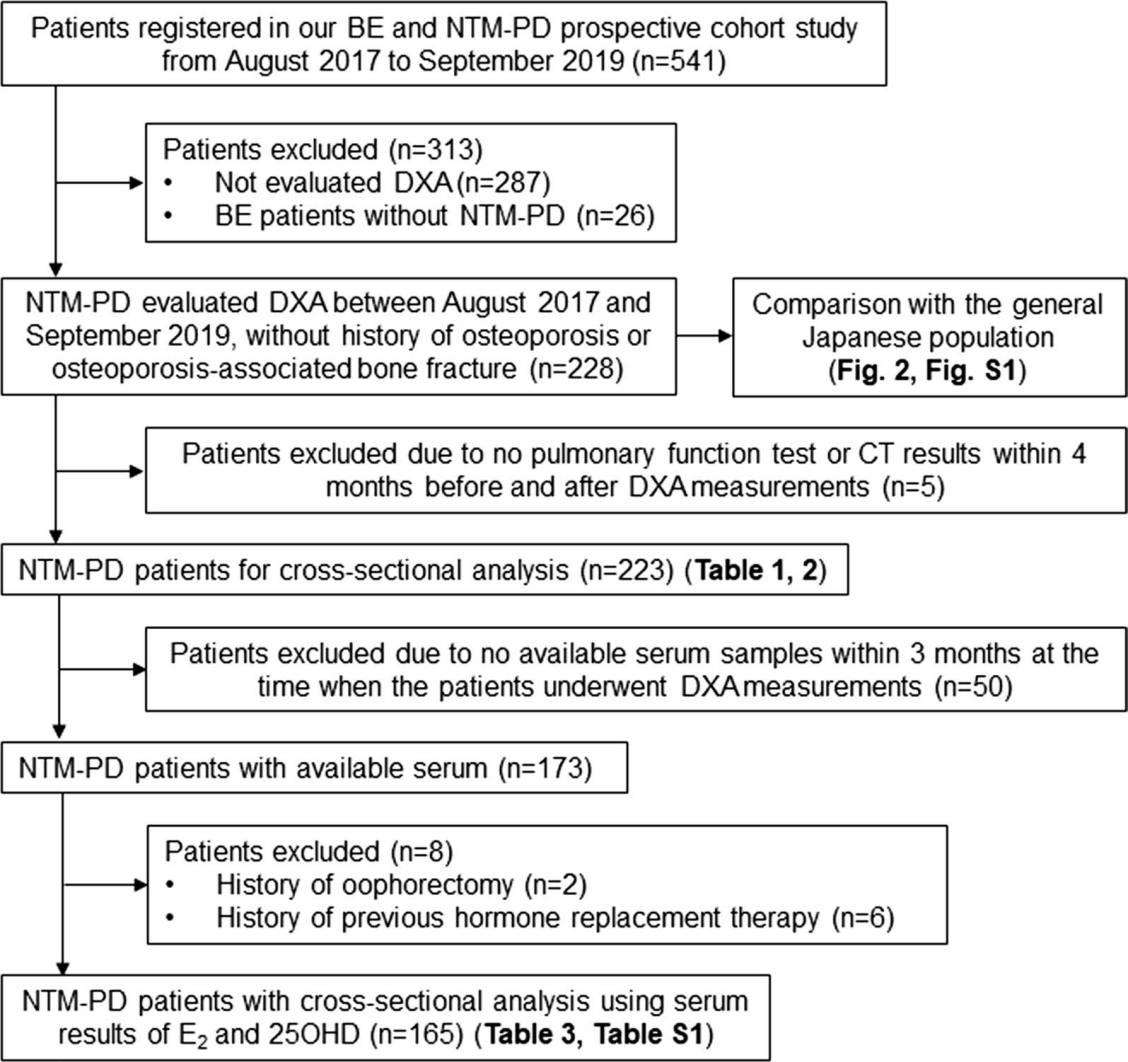
An enrollment process of the study

A cross-sectional study was conducted using the registry of a prospective cohort study that included patients with NTM-PD at Keio University Hospital [20–23]. NTM-PD was diagnosed based on the 2007 American Thoracic Society /Infectious Disease Society of America (ATS/IDSA) guidelines [8]. The Keio University Hospital ethics review board approved our study protocol (#20110267), and we obtained written informed consent from all eligible patients before their inclusion in the study. Figure 1 shows the enrollment process. Between August 2017 and September 2019, dual-energy X-ray absorptiometry (DXA)-based BMD was evaluated in 228 consecutive patients with NTM-PD, who had no history of osteoporosis or osteoporosis-associated bone fracture. The proportion of osteoporosis in the 228 patients was compared with that in the general Japanese population. Five patients did not undergo pulmonary function test (PFT), or multi-detector-row computed tomography (CT) within 4 months before and after DXA measurements. Therefore, we included 223 patients for the cross-sectional analysis.

We used stored serum samples previously obtained from the “NTM Biomarker Study” in the Keio University hospital (#20170181) to investigate the association of E_2_ and 25OHD with osteopenia/osteoporosis [11]. Available serum samples within 3 months at the time when the patients underwent DXA measurements were included and analyzed. Stored serum samples were available for 173 patients with NTM-PD. Eight patients with a history of oophorectomy or previous hormone replacement therapy were excluded to eliminate any possible medical effects. Finally, serum E_2_ and 25OHD were measured in 165 patients.

### Assessment of clinical parameters

We obtained patient demographic data, including age, sex, body mass index (BMI), disease duration, smoking history, underlying pulmonary and non-pulmonary diseases, current pharmacological treatment, treatment status for NTM-PD, and sputum smear and culture results of the previous year. Immunosuppressive or biological agents were defined as oral corticosteroids, calcineurin inhibitors, methotrexate, or any biological agents. We obtained patient data regarding chronic *Pseudomonas aeruginosa* (PA) infection as PA infection is known as a poor prognostic factor for bronchiectasis [24]. Chronic PA infection was defined as PA isolated from sputum culture on two or more occasions ≥ 3 months apart in any 1-year period in the past [20].

PFT was performed in a stable condition using an electronic spirometer (Chestac-9800 or HI-801; Chest M.I., Tokyo, Japan) according to the ATS/ERS recommendations [25]. PFT included the assessment of forced vital capacity (FVC) and forced expiratory volume in 1 s (FEV_1_). The radiographic CT patterns were categorized as nodular/bronchiectatic (NB), fibrocavitary (FC), NB + FC, or unclassified [26]. The severity of bronchiectasis was determined using the modified Reiff score [27]. The number of lobes involved (including the lingua, total six) and the degree of dilatation (tubular = 1, varicose = 2, and cystic = 3) were calculated with scores ranging from 0 to 18. Only the completed 6-min walk distance (6MWD), one of the important parameters associated with health-related quality of life in MAC-PD [28], was analyzed.

### DXA measurements

DXA measurements of BMD were performed at the hip and lumbar spine using a Hologic 4500A Discovery bone densitometer (HOLOGIC, Bedford, MA, USA). Osteoporosis was diagnosed based on the lowest T-score of these locations and World Health Organization criteria [29]. T score ≥ − 1, T score − 1 to − 2.5, T score ≤ − 2.5 were defined as normal BMD, osteopenia, and osteoporosis, respectively.

### ***Serum E***_***2***_*** and 25OHD levels***

The serum E_2_ and 25OHD levels were measured using a chemiluminescent immunoassay (CLIA) and electrochemiluminescent immunoassay, respectively, Since the serum E_2_ level under the limit of detection for CLIA was < 10 pg/mL, and because this level was associated with low BMD in postmenopausal women [30], low serum E_2_ levels were defined as < 10 pg/mL. Serum 25OHD levels were divided into normal (> = 30 ng/ml), insufficiency (< 30 ng/ml and >  = 20 ng/ml), and deficiency (< 20 ng/ml) [31].

### The general Japanese population

To compare the prevalence of osteoporosis in patients with NTM-PD in our study with that of the general population, we used data from the study by Yoshimura et al. [32] and calculated the proportion of osteoporosis in each age strata (≤ 39, 40–49, 50–59, 60–69, 70–79, ≥ 80 years). Our study used T score ≤ -2.5 for the diagnosis of osteoporosis, but the study by Yoshimura et al. [32] used a value of less than 70% of the Young Adult Mean (YAM). Another report (using T score ≤ − 2.5 in the same population) showed similar or rather a slightly lower rate of osteoporosis [33], but the raw data based on age and sex were not available to us. Additionally, another report showed that the T score and the YAM criteria were almost equivalent [34]. Therefore, the difference in criteria did not affect the results of the comparison between the two populations.

### Statistical analysis

We compared the prevalence of osteoporosis between patients with NTM-PD and the Japanese general population by Fisher’s exact probability test. To identify the factors associated with osteopenia/osteoporosis, we first checked multicollinearity in all the variables using Spearman’s correlation. We then performed multivariable logistic regression analyses using a backward elimination method based on the corrected Akaike information criterion (AICc) [35], starting with a model that included age, sex, BMI, smoking history, underlying pulmonary disease, connective tissue diseases, NTM-PD duration, treatment history of NTM-PD, acid-fast bacilli (AFB) smear positivity, NTM species (MAC or non-MAC), chronic PA infection, cavitary lesions, modified Reiff score, and % predicted FEV_1_ (%FEV_1_). In 165 NTM-PD patients whose E_2_ and 25OHD were measured, these variables were added for the multivariable analysis in the same manner. *P*-values were two-tailed, and *P* < 0.05 was considered statistically significant. Data were analyzed using the JMP 14 program (SAS Institute Japan Ltd, Tokyo, Japan). A graph of existing data was made with GraphPad Prism 8 (GraphPad Software, San Diego, California)***.***

## Results

### Comparison with the general population

**Fig. 2:**
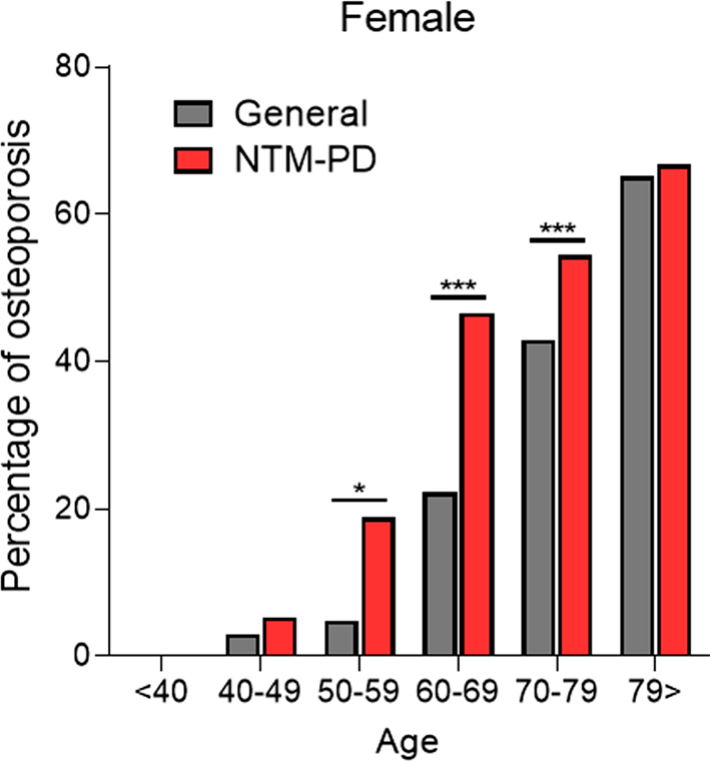
Comparison of the percentage of osteoporosis stratified by age group between female patients with nontuberculous mycobacterial pulmonary disease and in the Japanese general population [32]. **P < 0.01. ***P < 0.001

A total of 228 patients were included: 64 patients with normal BMD (28.1%), 84 with osteopenia (36.8%), and 80 with osteoporosis (35.1%). Figure 2 and Additional File 1: Figure S1 shows the comparison of the percentage of osteoporosis between the standard population [32] and that in female and male patients with NTM-PD, stratified by age group, respectively. The comorbidity was significantly higher in women with NTM-PD at age groups 50–59 years (18.8 vs. 4.8%, *P* = 0.010), 60–69 years (46.6 vs. 22.2%, *P* < 0.001), and 70–79 years (54.4 vs. 42.9%, *P* < 0.001).

### Patient characteristics

Table 1 shows the clinical characteristics of the 223 patients for the cross-sectional analysis, which included 64 patients with normal BMD (28.7%), 82 with osteopenia (36.8%), and 77 with osteoporosis (34.5%) The median age of the 223 patients was 70 years, and 181 patients were female (82.5%). The median duration of NTM-PD was 10 years. The most common causative species for NTM was MAC (204 patients, 91.5%), followed by *M. abscessus* complex (24 patients, 10.8%). Twenty-nine patients (13.0%) had chronic PA infection. The most common radiographic type was NB type (182 patients, 81.6%).

**Table 1:** Characteristics of the study population

	All patients (n = 223)	Normal BMD (n = 64)	Osteopenia (n = 82)	Osteoporosis (n = 77)
Age, years	70 (62–76)	66 (51–76)	69 (61–75)	73 (69–78)
Sex, female	181 (82.5)	42 (65.6)	69 (84.1)	73 (94.8)
BMI, kg/m^2^	19.3 (17.4–21.4)	20.2 (19.0–22.5)	19.2 (17.6–21.1)	17.9 (16.3–20.1)
Disease duration, years	10 (6–15)	9 (5–14)	9 (6–14)	11 (8–17)
Treatment history				
Never treated	87 (39.0)	26 (40.6)	35 (42.7)	26 (33.8)
Previously treated	25 (11.2)	9 (14.1)	8 (9.8)	8 (10.4)
Currently treated	111 (49.8)	29 (45.3)	39 (47.6)	43 (55.8)
Smoking status				
Not current / Current	221 (99.1) / 2 (0.9)	63 (98.4) / 1 (1.6)	81 (98.8) /1 (1.2)	77 (100) / 0 (0)
Underlying pulmonary disease	25 (11.2)	7 (10.9)	9 (11.0)	9 (11.7)
History of TB	6 (2.7)	4 (6.3)	2 (2.5)	0 (0)
Asthma	2 (0.9)	0 (0)	2 (2.5)	0 (0)
COPD	2 (0.9)	1 (1.6)	1 (1.3)	0 (0)
Interstitial lung disease	5 (2.3)	2 (3.2)	3 (3.8)	0 (0)
Comorbidities^*^				
Diabetes Mellitus	23 (10.3)	6 (9.4)	10 (12.2)	7 (9.1)
Chronic kidney disease	0 (0)	0 (0)	0 (0)	0 (0)
Connective tissue disease	15 (6.7)	3 (4.7)	7 (8.5)	5 (6.5)
Use of immunosuppressive or biological agents	11 (4.9)	3 (4.7)	4 (4.9)	4 (5.2)
Bacterial variables				
NTM species				
MAC	204 (91.5)	56 (87.5)	74 (90.2)	74 (96.1)
* M. abscessus* complex	24 (10.8)	8 (12.5)	8 (9.8)	8 (10.4)
* M. kansasii*	4 (1.8)	0 (0)	2 (2.4)	2 (2.6)
* M. fortuitum*	3 (1.3)	1 (1.6)	0 (0)	2 (2.6)
Sputum AFB smear positive^†^	82 (36.8)	19 (29.7)	26 (31.7)	37 (48.1)
Sputum AFB culture positive^†^	120 (53.8)	35 (54.7)	43 (52.4)	42 (54.5)
Chronic *P. aeruginosa* infection	29 (13.0)	4 (6.3)	10 (12.2)	15 (19.5)
Pulmonary function test				
FVC, L	2.52 (2.07–2.95)	2.97 (2.52–3.50)	2.55 (2.12–2.91)	2.12 (1.68–2.59)
FVC, % predicted	82.0 (70.8–94.6)	93.7 (78.7–120)	80.9 (71.5–91.7)	76.5 (64.7–86.3)
FEV_1_, L	1.79 (1.49–2.16)	2.22 (1.77–2.55)	1.78 (1.50–2.03)	1.58 (1.24–1.91)
FEV_1_, % predicted	71.2 (60.9–85.6)	79.9 (65.5–104)	70.0 (63.5–79.1)	67.9 (55.6–80.0)
FEV_1_/FVC	72.1 (67.2–78.4)	73.3 (67.9–78.3)	70.8 (65.6–76.2)	72.1 (67.8–78.5)
CT findings				
Radiographic type				
NB	182 (81.6)	48 (75.0)	67 (84.8)	64 (83.1)
FC	5 (2.2)	2 (3.1)	2 (2.5)	1 (1.3)
NB + FC	17 (7.6)	4 (6.3)	4 (5.1)	9 (11.7)
Unclassified	19 (8.5)	10 (15.6)	6 (7.6)	3 (3.9)
Presence of cavitary lesion	47 (21.1)	8 (12.5)	15 (18.3)	24 (31.2)
Modified Reiff score	4 (2–6)	3 (2–4)	3 (2–5)	5 (3–6)
Number of affected lobes	4 (3–5)	3 (2–4)	4 (3–5)	4 (4–6)
Six-min walk distance, m^‡^	450 (402–500)	465 (428–515)	450 (415–498)	415 (355–480)

Data are as N (%) or median (interquartile range)

*Abbreviations*: *AFB* Acid-fast bacilli, *BMI* Body mass index, *COPD* Chronic obstructive pulmonary disease, *FC* Fibrocavitary, *FEV*_*1*_ Forced expiratory volume, *FVC* Forced vital capacity, *MAC* *Mycobacterium avium* complex, *NB* Nodular/bronchiectatic, *NTM* Nontuberculous mycobacteria, *TB* Tuberculosis

^*^There are no patients with chronic kidney disease

^†^Bacterial status within the previous 1 year

^‡^Six-minute walk test was performed on 201 patients (61; normal BMD, 73; osteopenia, 67; osteoporosis)

**Fig. 3:**
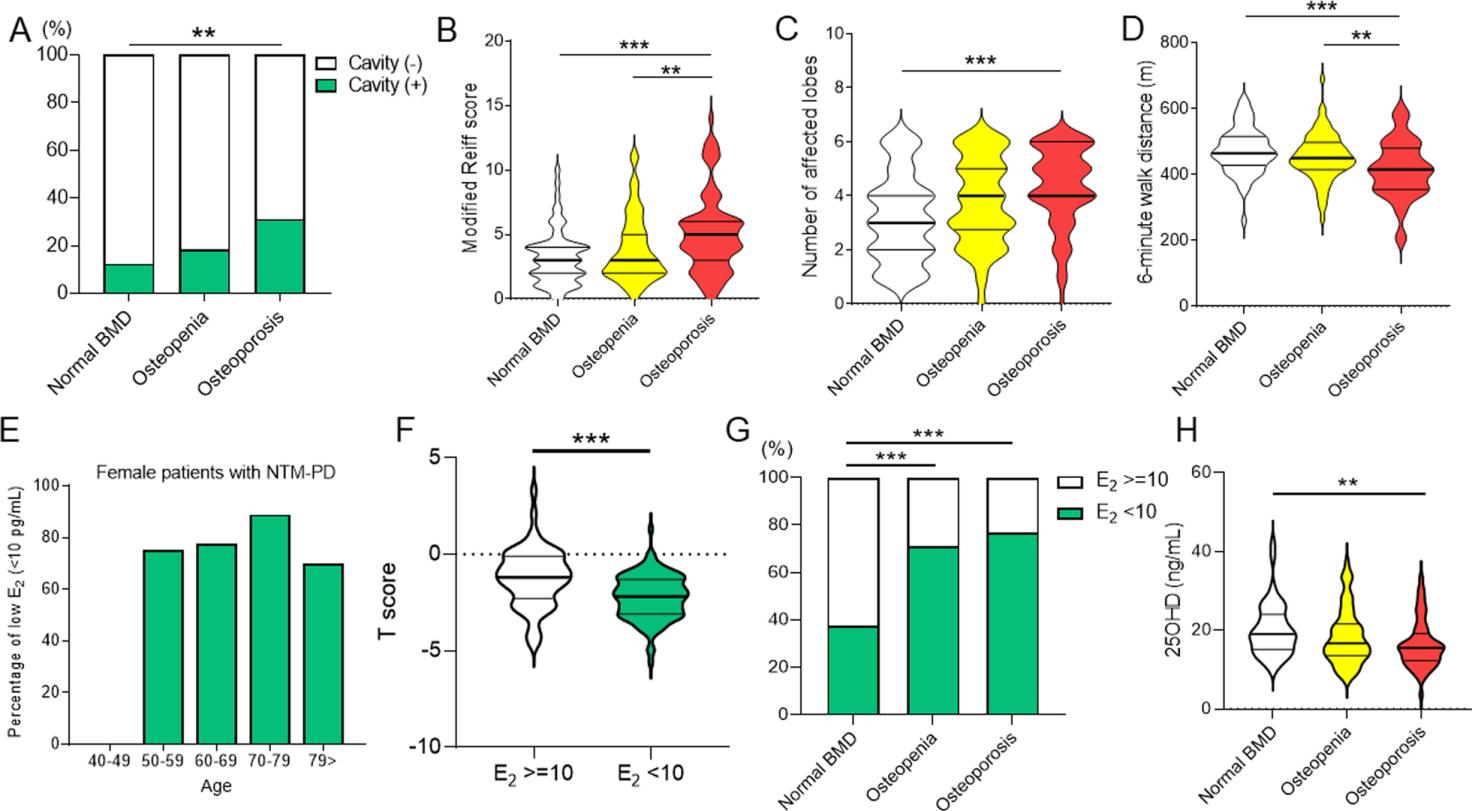
[A–D] Comparison of the cavitary lesion (**A**), modified Reiff score (**B**), number of affected lobes (**C**), and the 6-min walk distance (**D**) among the normal bone mineral density (BMD), osteopenia, and osteoporosis groups. **E** Percentage of low serum estradiol (E_2_) levels (< 10 pg/mL) stratified by age in female patients with nontuberculous mycobacterial pulmonary disease. **F** T-score compared between low serum E_2_ and others. **G** Comparison of low serum E_2_ status among normal bone mineral density (BMD), osteopenia, and osteoporosis groups. **H** Serum 25OHD levels represented as a continuous variable among normal bone mineral density (BMD), osteopenia, and osteoporosis groups. **P < 0.01. ***P < 0.001

In the comparison of patients with NTM-PD among the normal BMD, osteopenia, and osteoporosis groups, it was found that osteoporosis patients were older, predominantly females, and had a lower BMI. The PFT results revealed that FVC and FEV_1_ were lower in the osteopenia and osteoporosis group compared with those in the normal BMD group. Furthermore, the PFT results in the osteoporosis group were lower than those in the osteopenia group. The proportion of cavitary lesions, the modified Reiff score, and the number of affected lung lobes were higher in the osteoporosis group compared with the normal BMD group (Fig. 3A–C). The 6MWD was lower in the osteoporosis group compared with the normal BMD and osteopenia group but was comparable between the normal BMD and osteopenia groups (Fig. 3D).

Additional File 1: Table S1 shows a comparison of the characteristics of the 165 patients with NTM-PD whose serum E_2_ and 25OHD levels were measured. The distribution of these patients is as follows: normal BMD (n = 45, 27.3%), osteopenia (n = 63, 38.2%), and osteoporosis (n = 57, 34.5%) groups. The comparisons among the three groups are almost consistent with Table 1. Although the serum E_2_ levels in all male participants were ≥ 10 pg/mL, those in most female subjects were < 10 pg/mL (50–59-year-old, 75.0%; 60–69-year-old, 77.5%; 70–79-year-old, 88.9%; 80–89-year-old, 70.0%), except for those in 40–49-year-old women (Fig. 3E). Low serum E_2_ (defined as < 10 pg/mL) was associated with low T-score (Fig. 3F) with a higher proportion of osteoporosis and osteopenia groups, compared with the normal BMD group (Fig. 3G and Additional File 1: Figure S2). Although the comparisons of 25OHD, stratified by serum 25OHD levels, were comparable (Additional File 1: Figure S3), the serum 25OHD level as a continuous variable was lower in the osteoporosis group than in the normal BMD group (Fig. 3H).

### Factors associated with osteopenia/osteoporosis

Table 2 shows the results of multivariable logistic regression analysis in the 223 patients with NTM-PD. The analysis revealed that age (adjusted odds ratio [aOR], 1.04; 95% confidence interval [CI], 1.01–1.08), female sex (aOR 4.69, 95%CI 1.81–12.1) and chronic PA infection (aOR 4.31, 95%CI 1.08–17.2) were predictors of osteopenia, while age (aOR 1.12, 95%CI 1.07–1.18), female sex (aOR 36.3, 95%CI 7.57–174), lower BMI (aOR for 1 kg/m^2^ decrease 1.37, 95%CI 1.14–1.65), and chronic PA infection (aOR 6.70, 95%CI 1.07–41.8) were factors associated with osteoporosis.

**Table 2:** Multivariable logistic regression analysis of factors associated with osteopenia and osteoporosis in 223 NTM-PD patients

Characteristics	Osteopenia			Osteoporosis		
	aOR	95% CI	*P* value	aOR	95% CI	*P* value
Age, years	1.04	1.01–1.08	0.014	1.12	1.07–1.18	< 0.001
Sex, female	4.69	1.81–12.1	0.001	36.3	7.57–174	< 0.001
Lower BMI, 1 kg/m^2^ decrease	1.12	0.99–1.26	0.079	1.37	1.14–1.65	< 0.001
Chronic PA infection	4.31	1.08–17.2	0.039	6.70	1.07–41.8	0.042
Presence of cavitary lesion	–	–	–	2.39	0.74–7.72	0.145

Abbreviations: *aOR* Adjusted odds ratio, *BMI* Body mass index, *CI* Confidence interval, *NTM-PD* Nontuberculous mycobacterial pulmonary disease, *PA* *P. aeruginosa*

Table 3 shows the results of multivariate analysis in the 165 patients with NTM-PD whose serum E_2_ and 25OHD levels were measured. Low serum E_2_ (aOR 4.54, 95% CI 1.97–10.5) was the only predictor of osteopenia, while age (aOR 1.12, 95% CI 1.05–1.19), lower BMI (aOR for 1 kg/m^2^ decrease: 1.26, 95% CI 1.02–1.57), lower % FEV_1_ (aOR for 1% decrease: 1.04, 95% CI 1.01–1.07), low serum E_2_ (< 10 pg/mL)(aOR 3.29, 95% CI 1.01–10.7), and lower serum 25OHD (aOR for 1 ng/mL decrease: 1.12, 95% CI 1.03–1.23) were associated with osteoporosis.

**Table 3:** Multivariable logistic regression analysis in 165 NTM-PD patients whose serum E_2_ and 25OHD were measured

Characteristics	Osteopenia			Osteoporosis		
	aOR	95% CI	*P* value	aOR	95% CI	*P* value
Age, years	–	–	–	1.12	1.05–1.19	< 0.001
Lower BMI, 1 kg/m^2^ decrease	–	–	–	1.26	1.02–1.57	0.032
Chronic PA infection	3.49	0.61–19.9	0.159	–	–	–
Lower FEV_1_%predicted, 1% decrease	–	–	–	1.04	1.01–1.07	0.008
Low E_2_ (< 10 pg/mL)	4.54	1.97–10.5	< 0.001	3.29	1.01–10.7	0.048
Lower 25OHD, 1 ng/mL decrease	–	–	–	1.12	1.03–1.23	0.011

*Abbreviations* *25OHD* 25-hydroxyvitamin D, *aOR* Adjusted odds ratio, *BMI* Body mass index, *CI* Confidence interval, *E*_*2*_ Estradiol, *FEV*_*1*_ Forced expiratory volume in 1 s, *NTM-PD* Nontuberculous mycobacterial pulmonary disease, *PA* *P. aeruginosa*

## Discussion

Our study showed that 35.1% and 36.8% of patients with NTM-PD were newly diagnosed with osteoporosis and osteopenia, respectively, indicating that BMD loss in many patients with NTM-PD was unrecognized in clinical practice. Additionally, the proportion of osteoporosis in female patients with NTM-PD in the age groups 50–59, 60–69, and 70–79 years was significantly higher than that in the general population. Furthermore, factors including age, being female, lower BMI, and chronic PA infection were associated with osteoporosis. Our multivariable analysis revealed a significant association of age, being female and chronic PA infection with osteopenia. Finally, our multivariable analysis of patients whose serum E_2_ and 25OHD levels were measured revealed significant associations of both low serum E_2_ and 25OHD with osteoporosis. There are potentially a large number of patients with NTM-PD with untreated osteoporosis, and treatment may prevent decreased mobility and lung function due to fractures and falls. Bone density screening can improve the long-term prognosis and quality of life of patients with NTM-PD by increasing the intervention for undiagnosed osteoporosis.

To our knowledge, this is the first study revealing the prevalence of osteopenia/osteoporosis in patients with NTM-PD in a clinical setting, especially the higher proportion of osteoporosis in female patients with NTM-PD at 50–79 years. Importantly, our study showed that more than one third of patients with NTM-PD were underdiagnosed with osteoporosis. The risk factors for osteoporosis or bone fracture typically include age, female sex, low body weight, smoking, excessive alcohol intake, and glucocorticoid use [36]. BMI and glucocorticoid use, but not age and sex, have been associated with lower BMD in lung transplantation candidates [37]. Importantly, NTM-PD is more likely to occur in patients with these diseases that involve structural destruction of the lungs [16]. Our study revealed a higher prevalence of osteoporosis in female patients with NTM-PD at the stratified comparison of age and sex, indicating that other factors besides age and sex were also associated with osteoporosis.

Notably, the present study also revealed that lower serum E_2_ levels were associated with osteopenia and osteoporosis. The onset of osteoporosis is more common in the postmenopausal period with rapid depletion of estrogen, around the age of 50 years, because estrogen deficiency causes increased bone resorption and decreases bone density [38]. Further, in postmenopausal women, female hormone depletion has also been associated with a low BMI [39], which is also a major risk factor for osteoporosis and bone fracture [40]. Therefore, female hormone deficiency causes BMD loss directly and through decreased BMI. One of the NTM-PD subsets has been classically described as the Lady Windermere syndrome, as it occurs commonly in thin middle-aged/elderly women [41]. A previous report has shown that BMI and body fat are significantly lower in patients with NTM-PD [42]. Our previous study on postmenopausal women aged ≤ 65 years revealed that low serum E_2_ levels were strongly associated with MAC-PD, although, the menopausal age in both control and MAC-PD groups was around 50 years [11]. In addition, another study showed that median/mean serum E_2_ levels in normal postmenopausal Japanese women are more than 10 pg/mL [11, 43], while in our study, more than 70% of patients over 50 years had low serum E_2_ levels. A study in mice suggested that E_2_ may have a protective role against intracellular mycobacterial infection [44]. Additionally, low BMI has been associated with the development and severity of NTM-PD [45]. Taken together, female hormones and a low BMI are strongly associated with the development of NTM-PD, as well as with osteoporosis, and may have affected the high comorbidity of osteoporosis in the current study.

Our study revealed that chronic PA infection was associated with osteopenia and osteoporosis in addition to previously described factors such as age, sex, lower BMI, and lower serum E_2_ levels. Chronic PA infection has been associated with increased disease severity in adult bronchiectasis patients [24] and MAC-PD [20]. Our study showed that the osteoporosis group had a higher modified Reiff score, number of affected lobes, and lower lung function and 6MWD, all of which imply that this group contains has severe NTM-PD, causing immobilization. Thus, chronic PA might be associated with osteoporosis. Another possible cause is a negative pathophysiological effect of PA infection on osteoporosis. Basic studies have suggested that various proinflammatory cytokines, such as tumor necrosis factor-alpha (TNF-α) and receptor activator of NF-κB ligand (RANKL), which are released during infection, may promote the development and activity of osteoclasts [46]. A study on cystic fibrosis patients primarily colonized with PA showed that infective exacerbation induced an increased production of potential osteoclast precursors in the peripheral blood, which may increase bone resorption, contributing to BMD loss [47]. Although the direct effect of PA infection is still unclear, we should consider screening patients with severe NTM-PD, especially those complicated with chronic PA infection, for osteoporosis.

About 70% patients with NTM-PD showed vitamin D deficiency (< 20 ng/mL), which is similar to the results of a previous Korean study [12]. The lower serum 25OHD was independently associated with osteoporosis in NTM-PD. Vitamin D is essential for calcium absorption and can work importantly as an immunomodulator. Specifically, for mycobacterial infection, vitamin D deficiency was associated with tuberculosis progression [48, 49]. The osteoporosis group had higher severity of NTM-PD, which can induce immobilization and reduced exposure to sunlight can lower serum 25OHD; another possibility is that patients with low serum 25OHD might show disease progression as well as osteoporosis. Although, the causal relationship between serum vitamin D and NTM-PD is unclear, serum 25OHD may aid diagnosis with osteoporosis as well as vitamin D supplementation.

There are several limitations to the current study. First, though the population of NTM-PD at our institution was not significantly different from populations in other studies [50, 51], this is a single-center, cross-sectional study, which limits us to elucidate causal relationships. The paucity of underlying pulmonary diseases other than NTM-PD may also allow us to assess the direct impact of NTM-PD on BMD. Additionally, the general population we used included mountainous and seacoast patients outside of our institute in Tokyo. Therefore, an additional multicenter study would be desirable to avoid patient selection bias. Secondly, the small number of male patients included in the present study warrants additional evaluation. Future studies with a larger number of participants and analyses with gender and age matching are required. Third, the implementation of DXA in this cohort is based on the clinician's judgment of necessity, which could cause selection bias. We may have also underestimated the prevalence of osteoporosis in the study population because we excluded patients with previously noted osteoporosis. However, this study is retrospective, which is limited as a study design, and requires future prospective studies. Finally, the study did not measure any bone metabolism markers, which may provide more insight into the association and pathophysiology between pulmonary disease and osteoporosis. While we measured serum E_2_ and 25OHD, this showed a significant association between serum E_2_ and BMD loss, further longitudinal studies including these markers are needed to investigate the pathophysiology and effect of intervention including pharmacological treatment.

## Conclusions

The findings of our study revealed a higher incidence of osteoporosis in patients with NTM-PD than that in the general population of middle-aged/older women. BMD screening should be considered in patients with NTM-PD, especially when older in age, female, and diagnosed with severe diseases like chronic PA infection and lower BMI. The measurement of serum E_2_ and 25OHD levels may also be useful to assess the factors associated with osteopenia and osteoporosis in NTM-PD patients.

## Supplementary Information

**Additional file 1**: **Figure S1**. Comparison of the percentage of osteoporosis stratified by age group between male patients with nontuberculous mycobacterial pulmonary disease and in the Japanese general population. **Figure S2**. Serum estradiol (E2) level represented as a continuous variable among normal bone mineral density (BMD), osteopenia, and osteoporosis groups. Because the serum E2 level under the limit of detection for a chemiluminescent immunoassay was <10 pg/mL, all cases with E2 <10 pg/mL were equated with an E2 of 10 pg/mL. **Figure S3**. T-score stratified by serum 25-hydroxyvitamin D (25OHD) levels (normal ≥30 ng/ml; insufficiency <30 ng/ml and ≥20 ng/ml; deficiency <20 ng/ml) (**A**). Comparison of serum 25OHD status among normal bone mineral density (BMD), osteopenia, and osteoporosis groups (**B**). **Table S1**. Characteristics of the population in whom estradiol (E2) and 25-hydroxyvitamin D levels were measured.

## Data Availability

All data generated or analyzed during this study are included in this published article.

## Abbreviations

25OHD: 25-Hydroxyvitamin D
AFB: Acid fast bacilli
aOR: Adjusted odds ratio
BMD: Bone mineral density
BMI: Body mass index
CI: Confidence interval
COPD: Chronic obstructive pulmonary disease
DXA: Dual-energy X-ray absorptiometry
E_2_: Estradiol
FC: Fibrocavitary
FEV_1_: Forced expiratory volume in 1 s
FVC: Forced vital capacity
MAC: *Mycobacterium avium* Complex
MAC-PD: MAC pulmonary disease
NB: Nodular/bronchiectatic
NTM: Nontuberculous mycobacteria
NTM-PD: NTM pulmonary disease
PA: *Pseudomonas aeruginosa*
PFT: Pulmonary function test
TB: Tuberculosis
