# Supplementary material for: Osteoporosis in nontuberculous mycobacterial pulmonary disease: a cross-sectional study
Source: BMC Pulm Med. 2022 May 21;22:202. doi: 10.1186/s12890-022-01991-3 (PMC9123794; doi:10.1186/s12890-022-01991-3)
Supplement: Supplementary file 1 — Additional file 1: Figure S1. Comparison of the percentage of osteoporosis stratified by age group between male patients with nontuberculous mycobacterial pulmonary disease and in the Japanese general population. Figure S2. Serum estradiol (E2) level represented as a continuous variable among normal bone mineral density (BMD), osteopenia, and osteoporosis groups. Because the serum E2 level under the limit of detection for a chemiluminescent immunoassay was <10 pg/mL, all cases with E2 <10 pg/mL were equated with an E2 of 10 pg/mL. Figure S3. T-score stratified by serum 25-hydroxyvitamin D (25OHD) levels (normal ≥30 ng/ml; insufficiency <30 ng/ml and ≥20 ng/ml; deficiency <20 ng/ml) (A). Comparison of serum 25OHD status among normal bone mineral density (BMD), osteopenia, and osteoporosis groups (B). Table S1. Characteristics of the population in whom estradiol (E2) and 25-hydroxyvitamin D levels were measured. [file 12890_2022_1991_MOESM1_ESM.docx]

**[Additional File 1]**

**Supplementary Information**

**Osteoporosis in nontuberculous mycobacterial pulmonary disease: A cross-sectional study**

Hiromu Tanaka, Takanori Asakura, Shoji Suzuki, Satoshi Okamori, Tatsuya Kusumoto, Takunori Ogawa, Shunsuke Uno, Atsuho Morita, Ho Lee, Ho Namkoong, Hirofumi Kamata, Yasunori Sato, Yoshifumi Uwamino, Tomoyasu Nishimura, Makoto Ishii, Koichi Fukunaga, Naoki Hasegawa

| **[Additional File 1] Supplementary Table S1:**  Characteristics of the population in whom estradiol (E_2_) and 25-hydroxyvitamin D levels were measured | | | | |
| --- | --- | --- | --- | --- |
|  | **All patients (n = 165)** | **Normal BMD (n = 45)** | **Osteopenia (n = 63)** | **Osteoporosis (n = 57)** |
| Age, years | 70 (62-75) | 65 (53-76) | 70 (61-74) | 72 (69-75) |
| Sex, female | 136 (82.4) | 29 (64.4) | 53 (84.1) | 54 (94.7) |
| BMI, kg/m^2^ | 19.3 (17.4-21.0) | 19.9 (19.0-22.2) | 19.5 (17.7-21.0) | 18.1 (16.0-20.1) |
| Disease duration, years | 10 (7-15) | 10 (7-15) | 9 (6-14) | 13 (8-17) |
| Treatment history |  |  |  |  |
| Never treated | 61 (37.0) | 16 (35.6) | 27 (42.9) | 18 (31.6) |
| Previously treated | 19 (11.5) | 8 (17.8) | 4 (6.3) | 7 (12.3) |
| Currently treated | 85 (51.5) | 21 (46.7) | 32 (50.8) | 32 (56.1) |
| Smoking status |  |  |  |  |
| Not current / Current | 163 (98.8)/2 (1.2) | 44 (97.8)/1 (2.2) | 62 (98.4)/1 (1.6) | 57 (100) /0 (0) |
| Bacterial variables |  |  |  |  |
| Sputum AFB smear positive^*^ | 63 (38.2) | 13 (28.9) | 22 (34.9) | 28 (49.1) |
| Sputum AFB culture positive^*^ | 92 (55.8) | 24 (53.3) | 34 (54.0) | 34 (59.6) |
| Chronic *P. aeruginosa* infection | 18 (10.9) | 2 (4.4) | 6 (9.5) | 10 (17.5) |
| Pulmonary function test |  |  |  |  |
| FVC, L | 2.52 (2.12-2.95) | 2.93 (2.48-3.54) | 2.56 (2.15-2.89) | 2.19 (1.68-2.57) |
| FVC, % predicted | 82.3 (71.0-94.0) | 91.6 (76.5-123) | 83.2 (74.9-94.3) | 76.5 (63.1-85.3) |
| FEV_1_, L | 1.78 (1.50-2.14) | 2.21 (1.75-2.51) | 1.81 (1.51-2.01) | 1.60 (1.25-1.86) |
| FEV_1_, % predicted | 70.7 (60.9-83.0) | 76.3 (65.5-105) | 70.6 (63.9-78.8) | 67.6 (55.6-77.6) |
| FEV_1_/FVC | 71.5 (66.3-77.8) | 73.3 (67.9-76.2) | 69.9 (65.5-75.2) | 71.5 (67.8-78.4) |
| CT findings |  |  |  |  |
| Radiographic type |  |  |  |  |
| NB | 137 (83.0) | 34 (75.6) | 55 (87.3) | 48 (84.2) |
| FC | 3 (1.8) | 1 (2.2) | 1 (1.6) | 1 (1.8) |
| NB+FC | 11 (6.7) | 3 (6.7) | 2 (3.2) | 6 (10.5) |
| Unclassified | 14 (8.5) | 7 (15.6) | 5 (7.9) | 2 (3.5) |
| Presence of cavitary lesion | 31 (18.8) | 4 (8.9) | 10 (15.9) | 17 (29.8) |
| Modified Reiff score | 4 (2-5) | 3 (2-4) | 3 (2-5) | 4 (2-6) |
| Number of affected lobes | 4 (3-5) | 3 (2-4) | 4 (3-5) | 4 (3-6) |
| Six-min walk distance, m^†^ | 450 (400-500) | 475 (431-514) | 450 (410-506) | 410 (361-470) |
| Serum E_2_ level |  |  |  |  |
| Low E_2_ (lower than 10 pg/mL) | 106 (64.2) | 17 (37.8) | 45 (71.4) | 44 (77.2) |
| Serum 25OHD |  |  |  |  |
| Serum 25OHD level, ng/mL | 16.4 (13.6-21.8) | 19.0 (15.2-24.1) | 16.7 (13.5-21.6) | 15.5 (12.4-19.2) |
| Deficient (<20 ng/ml) | 113 (68.5) | 27 (60.0) | 41 (65.1) | 45 (78.9) |
| Insufficient  (<30 ng/ml and >=20 ng/ml) | 42 (25.5) | 15 (33.3) | 17 (27.0) | 10 (17.5) |
| Normal level (>=30 ng/ml) | 10 (6.1) | 3 (6.7) | 5 (7.9) | 2 (3.5) |
| Data are as N (%) or median (interquartile range).  Abbreviations: 25OHD, 25-hydroxyvitamin D; AFB, acid-fast bacilli; BMI, body mass index; E_2_, estradiol; FC, fibrocavitary; FEV_1_, forced expiratory volume; FVC, forced vital capacity; MAC, *Mycobacterium avium* complex; NB, nodular/bronchiectatic; NTM, nontuberculous mycobacteria.  ^*^Bacterial status within the previous 1 year.  ^†^6-min walk test was performed on 153 patients (44; normal BMD, 58; osteopenia, 51; osteoporosis). | | | | |


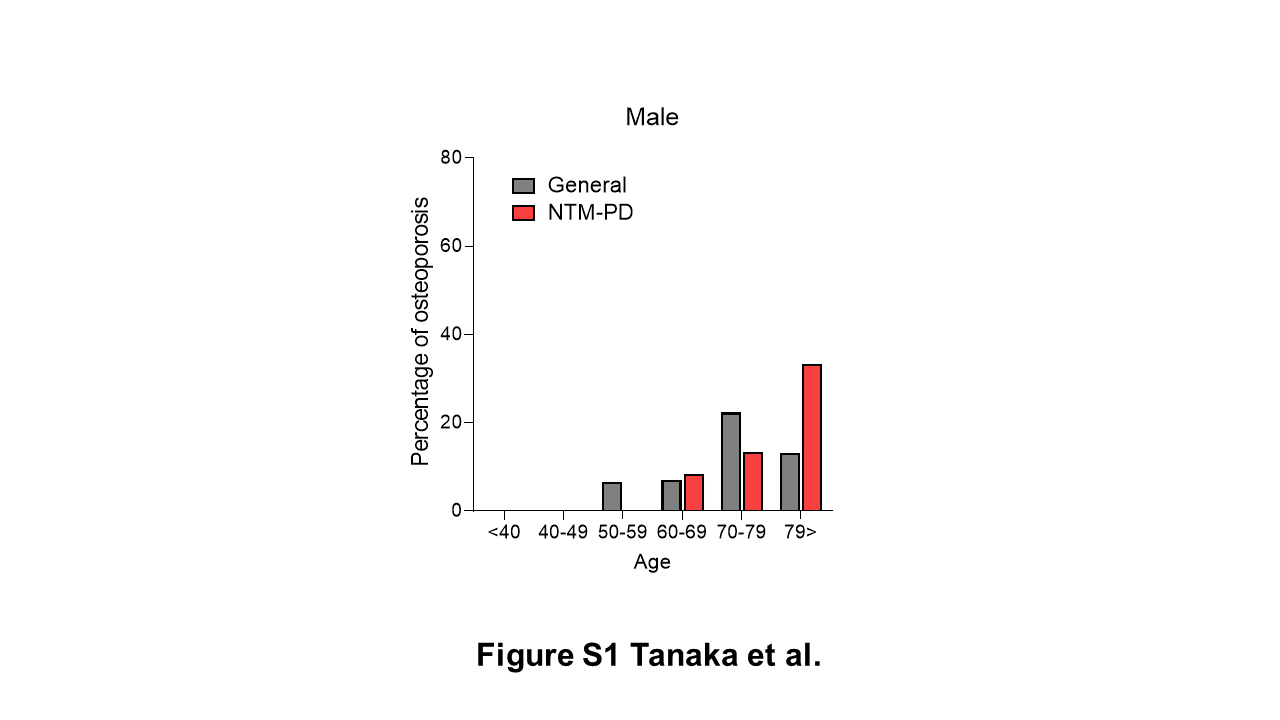


**[Additional File 1] Supplementary Figure S1**. Comparison of the percentage of osteoporosis stratified by age group between male patients with nontuberculous mycobacterial pulmonary disease and in the Japanese general population.

**[Additional File 1] Supplementary Figure S2**. Serum estradiol (E_2_) level represented as a continuous variable among normal bone mineral density (BMD), osteopenia, and osteoporosis groups. Because the serum E_2_ level under the limit of detection for a chemiluminescent immunoassay was <10 pg/mL, all cases with E_2_ <10 pg/mL were equated with an E_2_ of 10 pg/mL.


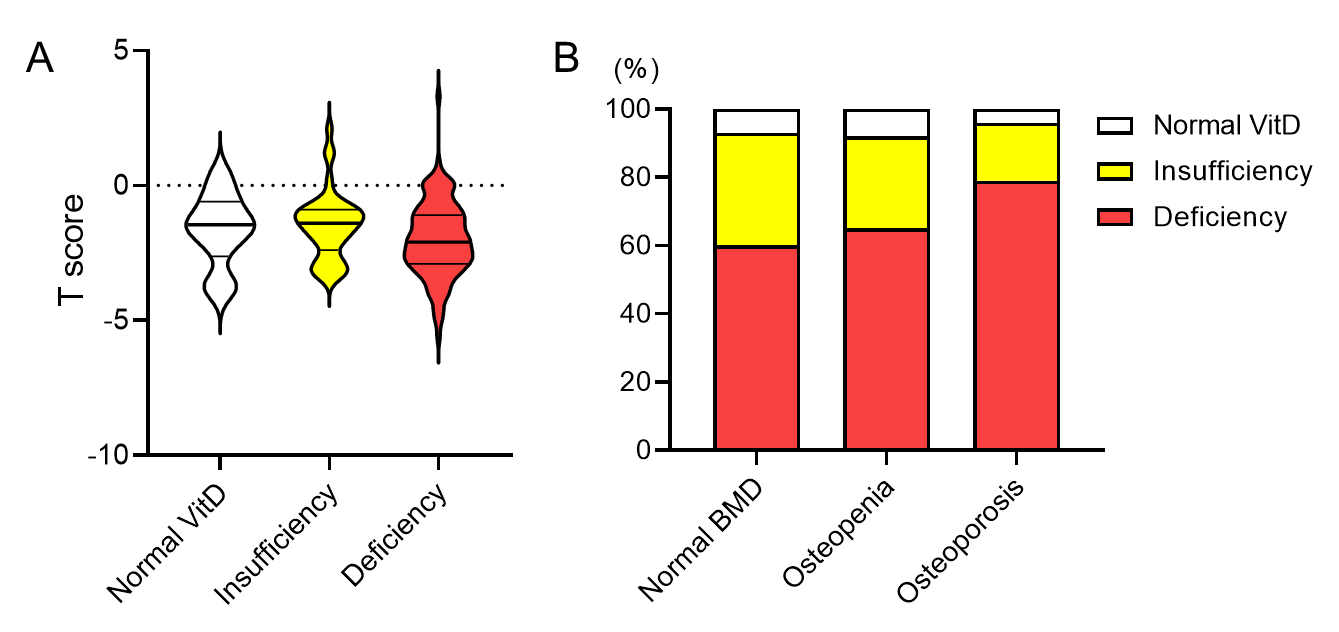


**[Additional File 1] Supplementary Figure S3**. T-score stratified by serum 25-hydroxyvitamin D (25OHD) levels (normal ≥30 ng/ml; insufficiency <30 ng/ml and ≥20 ng/ml; deficiency <20 ng/ml) (A). Comparison of serum 25OHD status among normal bone mineral density (BMD), osteopenia, and osteoporosis groups (B).
